# Can We Trust the Literature on Risk Factors and Triggers for Low Back Pain? A Systematic Review of a Sample of Contemporary Literature

**DOI:** 10.1155/2019/6959631

**Published:** 2019-05-12

**Authors:** Emad M. Ardakani, Charlotte Leboeuf-Yde, Bruce F. Walker

**Affiliations:** ^1^College of Science, Health, Engineering, and Education, Murdoch University, Murdoch, Australia; ^2^Institute for Regional Health Research, University of Southern Denmark, Odense, Denmark

## Abstract

**Background:**

Risk factors (RFs) for the “*disease*” of low back pain (LBP) are probably different from the triggers of new episodes of LBP. Investigating RFs for the onset of the “disease” and the triggers of LBP is problematic if researchers fail to discern the different types of pain-free status of participants at and before baseline. There is a difference between never having had LBP and having been pain-free for a certain period only. In this review, we assessed the dependability of contemporary literature on RFs and triggers of LBP, in relation to the “disease” and the episodes, respectively.

**Methods:**

A literature search from 2010 until 2017 was performed. Information on the definitions of LBP, potential RFs/triggers, and study design was extracted. Studies were reclassified based on the type of LBP concerning the “disease,” episode, or mixed/unclear/chronic. RFs and triggers were grouped into major domains, and positive associations listed, respectively, for the “disease” and episodes.

**Results:**

In 42 of the included 47 articles, it was not clear if the authors investigated RFs for the “disease” of LBP or triggers of new episodes. Only one study properly reported RFs for the onset of the “disease” of LBP, and four studies were deemed suitable to investigate triggers for a new episode of LBP. No study reproduced the results of other included studies.

**Conclusion:**

Trustworthy information regarding RFs and triggers of LBP is rare in the current literature. Future research needs to use precise definitions of LBP (onset of the “disease” vs. episodes) and nominate the timing of the associated factors in relation to the types of LBP as these are two critical factors when studying causes of LBP.

## 1. Introduction

Low back pain (LBP) is mostly defined as a symptom of unknown origin (so-called nonspecific LBP) [1] because there is often no apparent pathology detected. Even though it is difficult to study causes of an ailment when the pathology is not well understood, numerous research projects have been conducted trying to reveal what causes LBP. However, in doing this, researchers frequently do not separate the concepts of the underlying “disease” of LBP, namely, the onset of the very first episode, from its recurring episodes, that is, the continued manifestations of the “disease” [2]. This is important, as it is quite possible that causes differ for the onset of the “disease” itself, acknowledged as risk factors (RFs), and any subsequent causes of episodes, which should better be described as “triggers.” Therefore, when LBP is investigated for its RFs and triggers, it should be differentiated as the “disease” of LBP or as a mere episode of LBP. This differentiation depends on whether the study participant was completely “disease”-free prior to the onset of the symptoms or whether there had been several episodes and that there was a symptom-free period before the presently studied episode.

In this review, we report on contemporary research literature on this topic. Hence, we want to alert researchers to the need of providing a clear definition of LBP in relation to if it relates to the first onset of the underlying “disease,” or if it deals with the subsequent episodes of this “disease,” or if it is perhaps a mixture of two. Unsurprisingly, when a “disease”-free or pain-free period is not questioned properly at baseline, participants with chronic persistent LBP may also be included which adds more to the uncertainty.

We have previously shown in a systematic review that researchers approached this topic inappropriately [2]. The review demonstrated that, with few exceptions, authors do not separate (1) first-time, (2) episodic, and (3) ongoing LBP. Thus, it was revealed that, of the 33 articles, 31 seemed to have dealt with a mixture of two or even three types of LBP without a clear separation of these.

Since it is possible that these three types of LBP have different RFs and triggers, studies that do not distinguish between these different entities are at risk of not being able to provide meaningful answers to the question of causality. If we transfer this concept to a disease such as migraine, it will illustrate the difference between the RFs for the “disease” and triggers for episodes. While “stress” and “not eating” [3, 4] do not necessarily explain why some people develop the “disease” of migraine, these stimuli may trigger an episode of migraine headache. Similarly, triggers such as “sitting” or “lifting awkwardly” may explain the reappearance of episodes of LBP but not necessarily the primary RFs for the underlying “disease” of LBP.

In the present systematic review, we went further and aimed to highlight the consequences of this lack of definitions by concentrating on RFs and triggers studied in a sample of current studies. We hypothesised that, without this differentiation, many study participants have been asked to provide information on their personal life, physical activities, and psychosocial background, which have not contributed meaningfully to back pain science.

Our specific objectives were

1. To describe domains of associated factors that have been studied in a contemporary sample of the LBP literature from 2010 till 2017
2. To describe all RFs and triggers reported by authors for first-time ever LBP (onset of the “disease”) and a new episode of LBP, respectively
3. To appraise—based on our own precise classification—how useful contemporary literature is on true RFs for the first-time ever LBP and on the valid triggers of a recurring episode, and to make recommendations for any future research.

## 2. Methods

### 2.1. Search Strategy, Inclusion Criteria, and Exclusion Criteria

Articles from the first systematic review [5–37] were included (2010–2016) and a follow-up search using the same search parameters was performed to capture newer studies on this topic up until September 2017. The search strategy, criteria for inclusion and exclusion, and data extraction process were extensively described elsewhere (free full text online) [2]. Briefly, a literature search of the PUBMED, CINAHL, and SCOPUS databases was performed. The search period was selected arbitrarily to reflect a selection of contemporary literature. Articles containing the following keywords were included (low back pain OR back pain) AND (risk factor OR caus∗OR predict∗OR onset OR first-time OR inception OR incidence).

Studies were excluded if they were reviews, case reports, case-control, or reported exclusively on chronic/persistent LBP, specific cause LBP, secondary/tertiary care seekers, LBP in a special population (e.g., Parkinson's disease or pregnant women), and studies designed to investigate experimentally-induced LBP.

### 2.2. Data Extraction

Extraction of data was done independently by two reviewers, and disagreements in the data extraction forms were settled via discussion or by a third reviewer if it remained indecisive.

For the purposes of this review, we first classified the included studies based on what they were supposed to study according to their title, abstract, objectives, or methodology. Studies were assigned to three categories: (1) studies apparently dealing with a first-time episode (incidence of the “disease”), (2) studies allegedly investigating a new but recurring episode (incidence of a new episode), and (3) studies that likely included participants of mixed LBP definitions including ongoing chronic LBP (prevalence).

Then, using the stringent definitions described in our first systematic review, we identified which study actually fitted its claimed category based on the types of LBP studied. In more detail, if a study claimed or seemed to investigate the incidence of the “disease” of LBP, we reviewed the study to confirm whether participants had been clearly asked about being pain-free at baseline with no prior history of LBP. Moreover, if researchers claimed or seemed to study a new episode of LBP, we were interested to see if, at baseline, participants were distinctly identified with a prior pain-free period which was preceded by a previous episode. Specifically, we were interested to see if de Vet's proposed recommendation on defining a new episode was used. Her team recommended that an episode of LBP should last at least 24 hours and be preceded and followed by a period of at least 1 month without low back pain [38]. de Vet et al. appears to have been the first to highlight the necessity for a clear definition of an episode and nonepisode in order to bring unanimity into LBP research and make the interpretation of research findings more tangible. However, a relatively recent systematic review of the definitions of recurrent low back pain notes that there is still a great diversity in the definitions of recurrent LBP [39]. Nonetheless, only the validity of de Vet's definition of the duration of the pain-free period (a nonepisode) was studied in the general population and primary care and shown to be applicable [40, 41].

If a study did not fit one of these two groups, it was placed in the mixed category (which could include anything).

Thereafter, we listed all associated factors identified by the authors in each study and put them into different domains for the ease of reading. Examples of these domains are demographic (e.g., age, sex, and marital status), anthropometric (e.g., height, weight, and BMI), and lifestyle variables (e.g., smoking, drinking, leisure time, or habitual exercise). Factors that did not fit a specific domain were placed in “other.” The number of associated factors in each domain was also extracted for the first two LBP categories.

Finally, we identified studies that provided a minimum useful amount of causal information and reported which associated factors had been shown to be likely RFs or triggers for LBP, but only where the definition of LBP could be clearly elucidated. In this regard, we considered the following criteria to infer a causal relationship: (1) a correct definition of LBP, as discussed in our previous systematic review, (2) the temporal relationship of the association was correct, and (3) a significant association was demonstrated. When the results of a study met those criteria, we assumed that the authors had reported “true risk factors or triggers;” otherwise, it was considered an association.

## 3. Results

### 3.1. General Description of Studies

**Figure 1:**
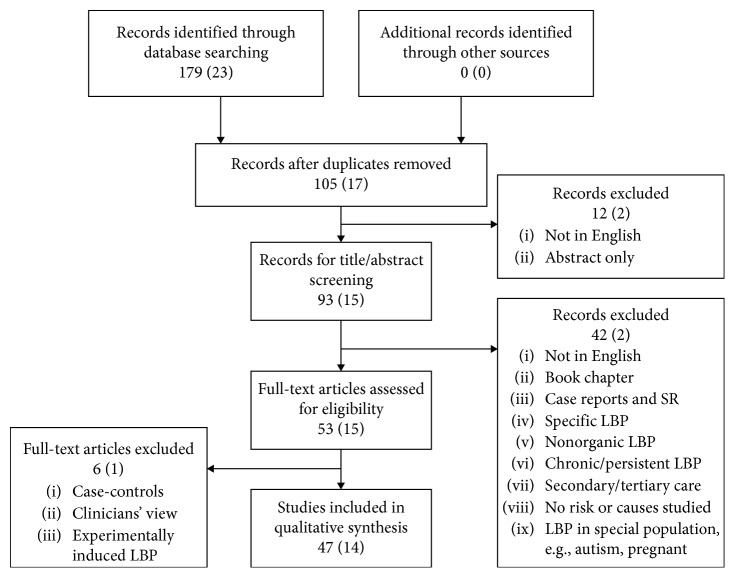
Flowchart of the search results (updated search results appeared in brackets).

For this review, 47 articles were included. Among those, 33 articles originated from our previous review [5–37] and 14 articles [42–55] were added after updating the search. The search process is shown in Figure 1.

A description of these articles is found in Table 1 and further summarised below.

Twenty studies were conducted in Asia/Middle East, 19 related to European countries/North America, 3 were from Australia and Africa, respectively, and 2 from South America.

Most included studies used a cross-sectional study design (*N* = 29, 62%), whereas the number of studies using a prospective design was 12 (26%). Other designs were retrospective (*N* = 5, 10%) and case-crossover (*N* = 1, 2%).

Although the authors indicated that they investigated risk factors for LBP, it was often unclear whether they targeted the “disease” or a subsequent episode of LBP. Therefore, based on what was previously described as our precise definition of LBP, we classified them into one of the three categories: (1) first-time episode, (2) recurring episode, or (3) mixed/unclear/chronic.

Five studies seemed to investigate the incidence of LBP [12, 15, 17, 28, 32]; but, we finally classified only one study as dealing with the true incidence of LBP [32], although in fact, this related to the first-time LBP caused by sports injury. In addition, even though the study design was retrospective, we found their findings credible since the RFs studied were obviously present prior to the very first episode of LBP (Table 2). However, the results may have been confounded as only bivariate analyses were used which may have limited the validity of the outcome.

Nine studies appeared to report on a new episode [7, 20, 21, 30, 34, 35, 43, 48, 49] (Table 3). Again, after having applied our rigorous definition of a recurrent episode, we were left with only one genuinely eligible article in the category [30], a study using a case-crossover design. Such designs are appropriate for studying triggers/exposures. Moreover, investigators clearly questioned the exposure to different triggers prior to each new episode. Nevertheless, 3 other studies [34, 35, 43] were finally eligible to be placed into the category of recurring episodes (further explained below). The investigators used a multivariate analysis of data to establish the statistical association in all four studies.

A major problem with the unacceptable studies was that they did not define the duration of the absence of LBP at baseline. LBP often starts in teenage years or even sooner [56–59], so theoretically, in the case of 5 of the studies, when authors recruited participants who were predominantly young [7, 20, 21, 48, 49], they could have included first-time episodes as well as recurring episodes. Such studies, therefore, are likely to have a mixture of study participants reporting on episodes and the “disease” of LBP.

However, since the study population in 3 other studies [34, 35, 43] were of adult age, it was less likely that the number of subjects with first-time LBP was high enough to significantly change the results. Therefore, we decided to include them in the recurring episode category. All of them applied a prospective design when collecting RF data.

Ultimately, 42 studies did not clearly belong to either the first-time episode or the recurring episode categories (Table 1).

### 3.2. General Description of Risk Factors/Triggers

It was not the purpose of this study to exhaustively identify RFs and triggers for LBP. The following information is provided to illustrate the small amount of information that was finally obtained relating to RFs for the “disease” of LBP or triggers of its episodes despite the large number of studies on this topic.

In the 47 studies included, the associated factors (possible RFs or triggers) reported could be grouped under 12 major headings with only a few not fitting into those groups (“other”). Examples of the latter were travelling with public transport, going to parties, and sexual activity (see Table 4 in the Supplementary Material for a comprehensive description of these domains).

**Figure 2:**
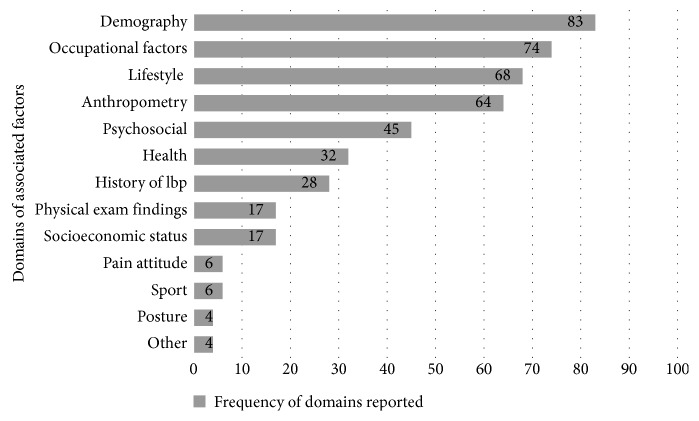
Domains of associated factors investigated in all included studies (regardless of the LBP definition).

Further scrutiny of all included studies, regardless of the categories in which we placed them, revealed a predominance for four domains, namely, demographic, occupational, lifestyle, and anthropometric factors. These four domains were tested for association with LBP in 83, 74, 68, and 64 percent of studies, respectively. On the other hand, the domains of pain attitude, posture, sport, and “other” were investigated in only 6 and 4 percent of studies. This is illustrated in Figure 2.

### 3.3. Risk Factors for the First-Time LBP

Based on our definitions of LBP, one study belonged to the category of first-time LBP [32]. In this study, RFs for the very first episode of LBP due to sports injury were found to be sex, fatigue, and some types of sport. To obtain these results, at least 16 individual RFs were tested (Table 2).

### 3.4. Triggers for a New Episode of LBP

Four studies were finally considered worthy of inclusion in the episodic LBP group. In these studies, we identified 51 variables that were studied as potential triggers of a new recurring episode of LBP, but only 8 were positively associated with the recurrence of a new episode. These were work-family imbalance, manual tasks, moderate and vigorous physical activity, distraction during a task, tiredness, history of LBP, poor mental health, and distressing somatic symptoms. One of the four studies was not able to identify any associated triggers from those studied [34] (Table 3).

## 4. Discussion

In this review, we aimed to find out how much information collected and reported as RFs or triggers could be trusted and genuinely provide evidence on LBP causality. A previous study had already revealed that most researchers failed to properly approach the question of LBP causality by not separating the “disease” and the episode [2]. This review, accordingly, showed that despite a large number of studies having been conducted on causes of pain in nonspecific LBP sufferers and a considerable amount of data collected, only a small fraction of the evidence is potentially trustworthy. Therefore, the consequence is a potential waste of a considerable amount of time, effort, and money—for both researchers and participants—without providing reliable answers about RFs or triggers for LBP.

This survey also demonstrates that a proper definition of LBP is even more crucial when the study population is young, as the mixture of first-time (inception) and recurring episodes is more probable as opposed to older adults, who are much less likely to experience LBP for the first time.

### 4.1. Causal Inference Requirements

What are the implications of this study on the causation of LBP? Causality, as used in epidemiologic studies, is the relation of causes to the effects they produce. Clearly, a cause must always precede an effect [60]. A RF is defined as “a factor that is causally related to a change in the risk of a relevant health process, outcome, or condition. The causal nature of the relationship is established on the basis of scientific evidence and causal inference” [60].

Association, by contrast, is “a mere statistical dependence between two or more events, characteristics, or variables. An association is present if the probability of occurrence of an event or characteristic, or the quantity of a variable, varies with the occurrence of one or more other events, the presence of one or more other characteristics, or the quantity of one or more other variables. The presence of an association does not necessarily imply a causal relationship” [60].

When investigating RFs for a disease, several requirements must be fulfilled before causality can be considered. One of them, which Bradford Hill in his famous article on causality identified, is the time sequence or temporal relationship of the association [61]. This is especially pertinent but may be difficult to determine in diseases that have long-lasting trajectories, such as LBP.

The concept that a RF must precede its disease and a trigger should precede a subsequent episode or exacerbation is a well-known tenet. However, a prospective study design does not automatically guarantee that the RFs or triggers (collected at study baseline) actually preceded the LBP (collected at follow-up). This is only true if the participant had never experienced LBP in the past (a requirement for the detection of the “disease”) or if the study subject experienced a limited LBP-free period at the time of baseline data collection (a requirement for an episode).

The majority of the included studies utilised a cross-sectional design. Due to the nature of this design, it is usually not possible to establish a temporal relationship between the suspected exposure and the outcome, and consequently, the causal inference is uncertain [62, 63]. However, it can sometimes be acceptable to draw inferential conclusions based on data from a cross-sectional design, as some nonmodifiable potential RFs would have preceded the onset of LBP regardless of in what stage of the “disease” cycle the study is carried out. Some examples of this are race, sex, parents' socioeconomic background, and family history of LBP.

Often, though, such risk factors and also some psychosocial triggers identified in this review such as work-family imbalance, poor mental health, and distressing somatic symptoms would not be real RFs or triggers, as they are unlikely to directly cause the “disease” of LBP or a new episode. Instead, there would be some hidden features in them that progressively predispose the individual to develop LBP. Therefore, it would be more appropriate to consider them “proxies” for risk factors or triggers.

### 4.2. Causal Links vs. Mere Associations

As mentioned above, it is well known that a significant association does not automatically imply causality [64, 65]. Hence, a critical feature of causality is the temporal relationship of the association [61]. Even though other Bradford Hill criteria of causation could have been examined in this review, we chose to focus on temporality because it is an absolute and fundamental criterion for causality and is particularly relevant in chronic diseases with multiple symptoms and phases such as LBP. It was not our intent to apply all of Bradford Hill's criteria in this review. In fact, it would be irrelevant to go any further, as long as the definition of the type of LBP is not clear.

Also, since chronic diseases can have multiple causative factors, a simple bivariate (by some called univariate) analysis, in which one RF is held up against the outcome variable, may not be sufficient. Authors, therefore, need to also consider how their data are best analysed when claiming a causal relationship. It is usually preferable to consider potential confounders and modifiers with other variables, which necessitates an insightful inclusion of other variables as well, i.e., various forms of multivariate analyses.

### 4.3. Methodological Consideration

We acknowledge that some weaknesses may have been present in this review. Despite the clarity of the search strategy, we cannot be certain that all relevant articles were retrieved. Also, our search period was limited to seven recent years. However, we deliberately intended to include only current literature, which could deliver an up-to-date understanding of how modern scientists deal with the issue of causality. It is possible that we did not identify all the articles on the topic. However, we were not uneasy about this, as our intention was not to determine RFs or triggers for LBP but to illustrate the “state of affairs” in this research domain.

Nonspecific back pain is a real back pain that cannot be diagnosed probably because the spine is a complex structure and many minor problems add up to one large one or because the pain is transient or because we simply do not understand what is going on. It seems though that whatever the underlying cause is, it is the muscles that compensate, so the pain is mainly felt in the back muscles. This does not mean that there is no underlying “disease.” Nonetheless, in our review, we could not take into consideration the possibility that some types of LBP possibly may change its type of “disease,” for example, by being aggravated or by spreading to other or a larger number of (unidentified) pain-carrying structures. Thus, it is possible that, along a lifetime, a person with LBP develops different “types” of LBP diseases, which may have different causes.

Data extraction was performed by two reviewers independently without any interest in the outcome. However, as the concept introduced by this review is relatively new, it was difficult to find relevant information from some studies. Despite that, on no occasion was a third reviewer required to solve the disagreement in data extraction, although it is of course still possible that this agreement occurred because both reviewers misinterpreted information in the same way. This systematic review was of an administrative nature and was not registered in PROSPERO.

## 5. Conclusion and Perspective

The results of this systematic review indicate that the vast majority of studies from the recent literature concerning RFs or triggers for LBP did not adequately differentiate first-time ever LBP (onset of the “disease”) and a new episode of LBP.

It is disappointing that regardless of the numerous studies carried out and a huge amount of information reported on this issue, the literature still lacks enough rigour to be useful and valid and to provide undisputed information on this topic. Thus, without such a simple criterion such as a proper definition of LBP, a considerable amount of time has been lost, both for many researchers and their study participants without adding meaningful information to the literature.

From a research perspective, it should now be clear that a precise definition of the prior pain-free period at baseline and consideration for the time sequence of the events are necessary elements to ponder for future research into the causality of LBP. From a clinical perspective, the lack of rigour around RFs and triggers research uncovered in this study leads to the unhappy conclusion that there is little preventive advice on back pain that can be provided at an individual level. From a public health perspective, this issue is crucial since nonspecific LBP is a very common and costly condition in the general population. Providers of funding, authors, and editors all share a common responsibility on this issue that of taking some basic definitions seriously, knowing that they impact heavily on the credibility of results.

## 6. Recommendations

The following recommendations from the results of the current review and our previous systematic review [2] are aimed at improving the credibility of future studies investigating causes of LBP and bringing more practical meaning and implications to their results. Study methodologies should

1. utilise the term “Risk factor” when investigating causes of the “disease” of LBP and “trigger” when studying causes of a recurring episode;
2. confirm a lifetime absence of LBP when investigating causes of the “disease” of LBP (i.e., the very first onset of LBP or incidence);
3. apply de Vet's definition of a nonepisode [38] or other published, described definitions in order to correctly identify a new recurring episode.

## Figures and Tables

**Table 1:** Characteristics of included studies on risk factors of LBP.

First author, year, country	Study design	Anticipated finding(s)^†^	First-time episode, episode, or prevalence^†‡^	Definition of preceding nonepisode^§^	“No prior history of LBP” queried	Type of LBP according to our definition
Anand, 2013, India [5]	Cross-sectional	Prevalence, associated risk factors	Prevalence	None	NA	Mixed/unclear
Alperovitch-Najenson, 2010, Israel [6]	Cross-sectional	Prevalence, associated risk factors	Prevalence	None	NA	Mixed/unclear
Auvinen, 2010, Finland [7]	Longitudinal	Risk factors	Episode	None	NA	Mixed/unclear
Capkin, 2015, Turkey [8]	Cross-sectional	Prevalence, risk factors	Prevalence	None	NA	Mixed/unclear
Cho, 2012, South Korea [9]	Cross-sectional	Prevalence, risk factors	Prevalence	None	NA	Mixed/unclear
Coenen, 2013, Netherlands [10]	Prospective cohort	Risk factor	Prevalence	None	NA	Mixed/unclear
Erick, 2014, Botswana [11]	Cross-sectional	Prevalence, risk factors	Prevalence	None	NA	Mixed/unclear
Ernat, 2012, North America [12]	Retrospective, database analysis	Incidence, risk factors	First-time	NA	Unclear	Mixed/unclear
Fajardo Rodriguez, 2016, Colombia [42]	Cross-sectional	Characterising LBP	Prevalence	None	NA	Mixed/unclear
Gaowgzeh, 2015, Saudi Arabia [13]	Cross-sectional	Prevalence, risk factors	Prevalence	None	NA	Mixed/unclear
Gold, 2017, North America [43]	Longitudinal	Predicting factors, risk factors	Episode	None	NA	Recurring episode^*∗*^
Hussain, 2017, Pakistan [44]	Cross-sectional	Prevalence, associated risk factor	Prevalence	None	NA	Mixed/unclear
Jia, 2016, China [14]	Cross-sectional	Prevalence, risk factors	Prevalence	None	NA	Mixed/unclear
Kanchanomai, 2015, Thailand [15]	Prospective	Annual incidence, risk factors for the onset	First-time	NA	No	Mixed/unclear
Katsavouni, 2014, Greece [16]	Cross-sectional	Risk factors	Prevalence	None	NA	Mixed/unclear
Kelley, 2017, North America [45]	Cross-sectional	Related factors	Prevalence	None	NA	Mixed/unclear
Kherad, 2016, Sweden [46]	Cross-sectional	Risk factors	Prevalence	None	NA	Mixed/unclear
Knox, 2014, North America [17]	Retrospective database analysis	Incidence, risk factors	First-time	NA	Unclear	Mixed/unclear
Labbafinejad, 2016, Iran [47]	Cross-sectional	Prevalence, risk factors	Prevalence	None	NA	Mixed/unclear
Lallukka, 2016, Finland [48]	Longitudinal	Determinant factors, risk	Episode	None	No	Mixed/unclear
Lin, 2014, Taiwan [18]	Cross-sectional	Prevalence, risk factors	Prevalence	None	NA	Mixed/unclear
Lin, 2012, Taiwan [19]	Cross-sectional	Prevalence, risk factors	Prevalence	None	NA	Mixed/unclear
Mattila, 2017, Finland [49]	Prospective	Predicting factor	Episode	None	No	Mixed/unclear
Mikkonen, 2016, Finland [20]	Prospective cohort	Risk	Episode	None	NA	Mixed/unclear
Mitchell, 2010, Australia [21]	Prospective	Factors predicting new-onset LBP	Episode	None	NA	Mixed/unclear
Mohd Anuar, 2016, Malaysia [50]	Cross-sectional	Prevalence, associated risk factors	Prevalence	None	NA	Mixed/unclear
Mohseni-Bandpei, 2011, Iran [22]	Cross-sectional	Prevalence, risk factor	Prevalence	None	NA	Mixed/unclear
Murtezani, 2011, Kosovo [23]	Cross-sectional	Prevalence, risk factors	Prevalence	None	NA	Mixed/unclear
Ng, 2014, Australia [24]	Cross-sectional	Prevalence, risk factors	Prevalence	None	NA	Mixed/unclear
Nissen, 2014, Denmark [25]	Retrospective	Risk factors	Prevalence	None	NA	Mixed/unclear
Noda, 2015, Sri Lanka [26]	Cross-sectional	Prevalence, risk factors	Prevalence	None	NA	Mixed/unclear
Rafeemanesh, 2017, Iran [51]	Cross-sectional	Prevalence, risk factors	Prevalence	None	NA	Mixed/unclear
Ramond-Roquin, 2015, France [27]	Prospective cohort	Risk factors	Prevalence	None	NA	Mixed/unclear
Shemory, 2016, North America [28]	Retrospective database analysis	Relative risk, incidence, risk factors	First-time	NA	None	Mixed/unclear
Sikiru, 2010, Nigeria [29]	Cross-sectional	Prevalence, risk factors	Prevalence	None	NA	Mixed/unclear
Şimşek, 2017, Turkey [52]	Cross-sectional	Prevalence, risk factors	Prevalence	None	NA	Mixed/unclear
Steffens, 2015, Australia [30]	Case-crossover	Risk factors	Episode	Yes	NA	Recurring episode
Sterud, 2013, Norway [31]	Prospective	Risk factors	Prevalence	None	NA	Mixed/unclear
Triki, 2015, Tunisia [32]	Retrospective	Prevalence, causes of injuries	First-time	NA	Yes	First-time episode
Udom, 2016, Thailand [53]	Cross-sectional	Prevalence, associated risk factors	Prevalence	None	NA	Mixed/unclear
Van Hilst, 2015, Netherlands [33]	Cross-sectional	Prevalence, associated risk factors	Prevalence	None	NA	Mixed/unclear
Vandergrift, 2012, North America [34]	Prospective	Risk factors, risk of incidence	Episode	None	NA	Recurring episode^*∗*^
Vargas-Prada, 2013, Spain [35]	Prospective	Factors predicting the incidence and prevalence	Episode	Yes	No	Recurring episode^*∗*^
Furtado, 2014, Brazil [36]	Cross-sectional	Risk factors	Prevalence	None	NA	Mixed/unclear
Yang, 2016, North America [54]	Cross-sectional	Prevalence, risk factors	Prevalence	None	NA	Mixed/unclear
Ye, 2017, China [55]	Cross-sectional	Risk factors	Prevalence	None	NA	Mixed/unclear
Yue, 2012, China [37]	Cross-sectional	Prevalence, risk factors	Prevalence	None	NA	Mixed/unclear

^†^According to authors or obvious from text: first-time episode: when the very first episode of LBP was investigated; episode: when any pain-free period preceding the recurrent episode under scrutiny could be identified; prevalence: when explicitly stated by authors or based on their objectives and method, a mixture of all types of LBP was investigated. ^§^An application of a pain-free period preceding the episode under scrutiny. ^*∗*^Were included in this category since the study population was mostly of an old adult age. NA: not applicable.

**Table 2:** Studies that seemed to study the onset of the “disease” of low back pain (first-time episode).

First author, year, country	Study design as defined by authors	Definition of LBP	Domains of associated factors investigated (number of included factors)	Types of analyses	Variables reported being linked to the incidence of LBP that were also credible RFs^†^
Ernat, 2012, North America [12]	Database analysis	Acute LBP resulting in an initial visit to a health care provider	Demographic (3)	Multivariate	Age
			Occupational factors (2)		Race
					Different types of military service

Kanchanomai, 2015, Thailand [15]	Prospective	Annual incidence of LBP >24 hrs in the past 3 months	Anthropometric (2)	Multivariate	Quadriceps muscle length
			Demographic (4)		
			Health (1)		
			Lifestyle (1)		
			Musculoskeletal examination findings (5)		Low back support while using a computer
			Psychosocial (1)		
			Occupational factors (1)		

Knox, 2014, North America [17]	Database analysis	First-occurrence LBP required seeking care	Demographic (3)	Multivariate	Sex
			Occupational factors (2)		Age
					Rank in military
					Type of service
					Marital status

Shemory, 2016, North America [28]	Database analysis	Medical diagnosis of LBP	Anthropometric (1)	Bivariate	None
Lifestyle (2)
Psychological (1)

Triki, 2015, Tunisia [32]^*∗*^	Retrospective cross-sectional	First-time LBP due to sports injury	Anthropometric (3)	Bivariate	Sex
			Demographic (2)		Fatigue (duration)
			Fatigue (1)		Some types of sport
			Lifestyle (1)	
			Type of sport (9)	

^†^Present before the onset of 1st-time episode and analysed taking into account also other variables. ^*∗*^The study truly investigated RFs based on our definition.

**Table 3:** Studies that seemed to investigate the onset of a new episode of low back pain (a recurring episode).

First author, year, country	Study design as defined by authors	Definition of LBP	Domains of associated factors investigated (number of included factors)	Types of analyses	Variables reported being linked to recent episode of LBP that were also credible triggers†
Auvinen, 2010, Finland [7]	Longitudinal	LBP at age 18	Anthropometric at 16 (2)	Multivariate	For girls, being too tired and insufficient sleep at 16
			Lifestyle habits at 16 (4)		For boys having 9 hrs sleep at 16
			Pain status at 16 (1)		
			Parents' socioeconomic status (1)		
			Psychological at 16 (3)		
Gold, 2017, North America [43]^*∗*^	Longitudinal	LBP past 3 months with at least mild severity during prior week	Anthropometric (1)	Multivariate	Work-family imbalance
			Demographic (5)		
			Health (2)		
			Lifestyle (2)		
			Occupational factors (5)		
Lalluka, 2016, Finland [48]	Longitudinal	Radiating LBP and local LBP lasting >7 days in the past year	Anthropometric (1)	Multivariate	Physical work heaviness
			Demographic (1)		
			Lifestyle (1)		
			Occupational factors (3)		
Mattila, 2017, Finland [49]	Prospective	Acute LBP (lumbago) in the last month. LBP radiating below knee in the last month	LBP during military service (1)	Multivariate	LBP during military service
			Maximal oxygen uptake (1)		
			Muscle fitness (6)		
Mikkonen, 2016, Finland [20]	Prospective cohort	Reporting new LBP at 18, Consultation for LBP at 18	Anthropometric at 16 (1)	Multivariate	Externalizing behaviour associated with consultation for LBP at 18 yrs in girls (not in boys)
			Lifestyle at 16 (4)		
			Parents' socioeconomic status at 16 (1)		
			Psychological at 16 (8)		
Mitchell, 2010, Australia [21]	Prospective	New significant LBP episode	Anthropometric (1)	Multivariate	History of LBP
			Demographic (1)		Stress
			Hx of LBP (1)		Moderate activity
			Lifestyle/Social (9)		Back muscle endurance
			Physical examination findings (5)		Pelvic angle slums sitting
			Posture (1)		Lower lumbar sitting repositioning error
			Psychological (4)		
Steffens, 2015, Australia [30]^*∗*^	Case-crossover	Sudden-onset acute LBP	Lifestyle (2)	Multivariate	Manual tasks
			Occupational factors (5)		Moderate and vigorous physical activity
			Psychosocial (3)		Distraction during a task
			Other (1)		Tiredness
Vandergrift, 2012, North America [34]^*∗*^	Prospective	More than 3 episodes of pain or one week pain in last 12 months	Anthropometric (3)	Univariate and multivariate	None
			Demographic (3)		
			Occupational factors (5)		
			Psychosocial (2)		
Vargas-Prada, 2013, Spain [35]^*∗*^	Prospective	LBP for a day or more in the past month	Demographic (3)	Multivariate	History of LBP
			Health beliefs about LBP (1)		Poor mental health
			History of LBP past 12 months (1)		Distressing somatic symptoms
			Lifestyle (1)		
			Mental health (1)		
			Occupational lifting (1)		
			Psychological (3)		
			Somatising tendency (1)		

^†^Present before the onset of a new recurring episode and analysed taking into account also other variables. ^*∗*^The study truly investigated triggers as per our definition.

## Supplementary Materials

Supplementary Materials Table 4 describes all associated factor domains studied by the included studies regardless of their LBP classifications.
