# Supplementary material for: Can We Trust the Literature on Risk Factors and Triggers for Low Back Pain? A Systematic Review of a Sample of Contemporary Literature
Source: Pain Res Manag. 2019 May 12;2019:6959631. doi: 10.1155/2019/6959631 (PMC6535889; doi:10.1155/2019/6959631)
Supplement: Supplementary Materials — Table 4 describes all associated factor domains studied by the included studies regardless of their LBP classifications. [file 6959631.f1.pdf]

Table 4. Associated factors domains studied in all included studies regardless of the LBP definition

**First author, Year, Country**	**Demographic**	**Socioeconomic**	**Anthropometric**	**Health**	**History of LBP**	**Physical examination findings**	**Posture**	**Lifestyle**	**Work ergonomics, Stress at work**	**Psychologic/ Psychosocial**	**Sport**	**Pain attitudes: Work/physical activities worsens pain, Pain attitudes in general**	**Other**
Aggarwal 2016 India								X	X	X		X	X
Alperovitch 2010 Israel				X				X	X	X			
Auvinen 2010 Finland		X	X		X			X		X			
Capkin 2015 Turkey	X	X		X	X			X					
Cho 2012 South Korea	X			X		X		X					
Coenen 2013 Netherlands	X				X			X	X				
Erick 2014 Australia	X		X					X	X	X			
Ernat 2012 North America	X								X				
Fajardo 2016 Colombia	X		X	X		X		X	X				
Gaowgzeh 2015 S Arabia	X			X	X				X				
Gold 2017 North America	X		X	X				X	X				
Hussain 2017 Pakistan							X		X	X			
Jia 2016 China	X		X					X	X	X			
Kanchanomai 2015 Thailand	X		X	X				X	X				

Katsavouni 2015 Greece	X		X					X	X		
Kelley 2017 North America	X				X				X		
Kherad 2016 Sweden	X	X	X	X	X			X			
Knox 2014 North America	X								X		
Labbafinejad 2016 Iran	X	X				X		X	X	X	
Lalluka 2016 Finland	X		X					X	X		
Lin 2012 Taiwan	X				X				X		
Lin 2014 Taiwan	X		X	X				X	X		
Mattila 2017 Finland					X	X					
Mikkonen 2016 Finland		X	X					X		X	
Mitchell 2010 Australia	X		X		X	X	X	X		X	
Mohd Anuar 2016 Malaysia	X		X						X		
Mohseni-Bandpei 2011 Iran	X		X	X				X	X		
Murtezani 2011 Kosovo	X		X					X	X		
Ng 2014 Australia	X		X								X
Nissen 2014 Denmark	X		X					X	X	X	

Noda 2015 Singapore	X	X	X				X	X	X			
Rafeemanesh 2017 Iran	X		X	X			X					
Ramond-Roquin 2015 France	X		X					X	X			
Shemory 2016 North America			X				X		X			
Sikiru 2010 Nigeria	X				X			X			X	
Simsek 2017 Turkey	X		X				X	X	X			
Steffens 2015 Australia							X	X	X			X
Sterud 2016 Norway	X							X	X			
Triki 2015 Tunisia	X		X			X	X			X		
Udom 2016 Thailand	X		X	X		X	X	X	X			
van Hilst 2015 Netherlands	X		X	X				X		X	X	
Vandergrift 2012 North America	X		X					X	X			
Vargas-Prada 2013 Spain	X			X	X		X	X	X			
Villar Furtado 2014 Brazil	X		X	X	X	X	X		X			
Yang 2016 North America	X	X	X				X	X	X			
Ye 2016 China	X	X	X		X			X				

Yue 2012<br>China	X	X		X	X
